# Impact of nursing shift patterns on work-related gastrointestinal disorders: a systematic review and meta-analysis

**DOI:** 10.3389/fpubh.2026.1839569

**Published:** 2026-06-05

**Authors:** David Pérez-Manchón, Cristina M. Lozano-Hernández, Gema Mata-González, Angel Jesús Arias-Arias, Noemí Mayoral-Gonzalo, Marina Gómez-de Quero Córdoba, Cayetana Ruiz-Zaldibar

**Affiliations:** 1 Department of Nursing, HM Faculty of Health Sciences, Camilo José Cela University, Villanueva de la Cañada, Madrid, Spain; 2 University Center for Health Sciences - HM Hospital (CUHMED), Camilo José Cela University, HM Hospital Health Research Institute, Madrid, Spain; 3 Department of Nursing, Physiotherapy and Occupational Therapy, Faculty of Nursing, University of Castilla-La Mancha, Albacete, Spain; 4 Institute for Health Research of Castilla-La Mancha (IDISCAM), Toledo, Spain; 5 Research Network on Chronicity, Primary Care and Health Promotion -RICAPPS-(RICORS), ISCIII, Barcelona, Spain; 6 Research Unit, Hospital Universitario Mancha Centro, Alcázar de San Juan, Spain; 7 Biomedical Research Network Centre for Liver and Digestive Diseases (CIBEREHD), Madrid, Spain; 8 Department of Nursing, Universitat Rovira i Virgili, Tarragona, Spain

**Keywords:** chronodisruption, gastrointestinal disorders, meta-analysis, nurses, shift work schedule

## Abstract

**Background:**

Gastrointestinal diseases are highly prevalent worldwide and are linked to poorer quality of life and increased use of healthcare services. Nursing shift work may disrupt circadian rhythms and contribute to functional gastrointestinal disorders through chronodisruption, sleep impairment, stress, and lifestyle changes. This review aims to analyze the relationship between nursing shift work and the development of gastrointestinal disorders.

**Methods:**

A systematic review and meta-analysis of different observational studies was conducted to evaluate associations between nurses' work shifts and gastrointestinal disorders. PROSPERO: CRD420251018324. An extensive search of the Databases (PubMed, Scopus, Web of Science, CINAHL) was carried out between April and May 2025 with no date restrictions applied. The studies included compared the frequency of gastrointestinal outcomes among >50% of practicing nurses working fixed day shifts or rotating shifts (including night work). Study quality was assessed using NOS (cohort/case-control) and JBI (cross-sectional). Risk of bias was evaluated with ROBINS-E. Odds ratios (OR) with 95% confidence intervals (CI) were pooled using fixed or random-effects models depending on heterogeneity.

**Results:**

Seventeen studies (data collection 1998–2023; publication 2003–2024) were included in the review, and 13 were incorporated into the meta-analysis. Most of the studies were cross-sectional and based in hospitals. Rotating shifts were associated with a significantly increased overall risk of gastrointestinal outcomes when compared to day shifts (OR = 1.15; 95% CI: 1.02–1.30; *p* = 0.02), with moderate heterogeneity (I^2^ = 53%). Subgroup analyses showed significant and robust associations for irritable bowel syndrome (OR = 1.74; 95% CI: 1.27–2.37; I^2^ = 0%) and diarrhea (OR = 2.41; 95% CI: 1.01–5.75; I^2^ = 0%). No significant associations were identified for colorectal cancer or inflammatory bowel disease. Most studies had low risk of bias, although outcome reporting and confounding were frequent concerns.

**Conclusion:**

Rotating shift work among nurses, particularly schedules including night shifts, is associated with a higher risk of gastrointestinal disorders, notably IBS and diarrhea. These findings support the need for occupational health strategies targeting chronodisruption, sleep quality, stress, and healthy lifestyle behaviors in shift-working nurses. They call for improved monitoring of gastrointestinal symptoms and further longitudinal research to identify long-term disease risks.

**Systematic review registration:**

https://www.crd.york.ac.uk/PROSPERO/view/CRD420251018324, identifier: CRD420251018324.

## 1 Introduction

Gastrointestinal diseases (GID) encompass pathologies affecting different anatomical segments of the digestive tract (1). The most prevalent GID can be classified into infectious enteric diseases (acute gastroenteritis), functional disorders (irritable bowel syndrome, inflammatory bowel disease, functional dyspepsia, constipation vs. non-infectious diarrhea), acid-peptic disease (dyspepsia, gastroesophageal reflux), liver diseases, and severe conditions such as gastrointestinal cancers (2).

In 2019, approximately 7.32 billion incident cases were reported worldwide (2), with an estimated 40% of the adult population affected (3). These conditions are associated with reduced quality of life and increased use of healthcare services. At the occupational level, functional bowel disorders, functional dyspepsia, and irritable bowel syndrome (IBS) are the most prevalent, leading to high rates of work absenteeism driven by chronic pain, fecal urgency, and fatigue (4).

In the hospital setting, continuous nursing care involves shift work, particularly rotating shifts. This is an inherent characteristic of the nursing profession and is essential to ensure 24 h continuous patient care (5). This work pattern may lead to desynchronization of circadian rhythms among these professionals, which has been associated with chronodisruption influencing visceral sensitivity and the circadian physiology of the gastrointestinal (GI) tract. This includes alterations in intestinal motility and increased nocturnal gastric acid secretion (6, 7), demonstrating a significant association with GID.

These GI disorders are independently and directly associated with factors such as circadian rhythm disruption, psychological stress, and poor sleep quality (8) among nurses with GID working rotating shifts compared to those working day shifts (9).

Irritable Bowel Syndrome (IBS) is one of the most common Functional Gastrointestinal Disorders (FGID) associated with shift work among nurses (10). Similarly, functional dyspepsia has a substantial impact related to sleep deprivation and psychological stress. General symptoms such as gastric pain or loss of appetite also affect nursing professionals (11).

To mitigate gastrointestinal impact on this occupational group, research on interventions related to shift work, particularly rotating shifts, suggests the need to improve sleep quality, stress management, and the promotion of healthy lifestyles, including diet and physical activity.

The aim of this study is to analyze the relationship between work shifts and the development of gastrointestinal disorders among nurses.

**Research question**: What is the association between shift work and the incidence of gastrointestinal diseases among nurses?

## 2 Materials and methods

### 2.1 Objectives

• To examine the most prevalent gastrointestinal diseases among nurses working shifts.

• To analyse the influence of fixed, morning, night, or rotating shifts on the development of gastrointestinal disorders.

### 2.2 Study design

This study is a systematic review and meta-analysis of studies that evaluated the relationship between nurses' shifts, categorized as day shifts (morning and afternoon: from 7:00 AM to 10:00 PM) and rotating shifts (shifts including night work), and gastrointestinal disease.

### 2.3 Selection criteria

The selection criteria were based on predefined criteria using PECOS (12) [Population, Exposure, Comparison, Outcome, Study Design] (Supplementary Table S1).

Inclusion criteria were as follows:

- Studies with samples of more than 50% practicing nurses.
- Studies that analyzed different nursing shifts and classified them as day or night shifts, for example: fixed day or night shifts, morning and afternoon shifts, or rotating shifts that included night shifts.
- Studies that analyzed gastrointestinal problems in nurses, such as irritable bowel syndrome, inflammatory bowel disease, constipation, diarrhea, gastric pain, and other related conditions.
- Studies that compared or analyzed gastrointestinal disorders in different shifts.
- Observational and descriptive studies, including cross-sectional, case-control, or cohort studies.

Exclusion criteria were as follows:

- Studies with a sample of less than 50% of practicing nurses or those focusing on student nurses.
- Studies that analyzed a single nursing shift and reported data on hours worked only.
- Studies focusing on the COVID-19 pandemic period.
- Review articles, intervention studies, and study protocols.
- Studies that are not published in Spanish or English.

No restrictions on age, gender, or publication date were applied.

### 2.4 Data sources

This study consulted the databases PubMed, Scopus, Web of Science (WOS) and CINHAL between April and May 2025. For the development of the search strategy, the Medical Subject Headings (MeSH) terms targeted three areas: nursing, shift work schedule, and gastrointestinal problems. The final search strategy combined these terms with the Boolean operators AND and OR (See Supplementary Table S2).

### 2.5 Study selection process

The search strategy described above was applied to the different databases. Duplicates were discarded and two researchers (DPM and CMLH) independently screened the titles and abstracts of all identified articles by applying the inclusion and exclusion criteria. During this process, a third researcher (CRZ) resolved discrepancies between the two to determine the studies eligible for full-text review.

In the subsequent selection phase, the initial two researchers independently assessed the full texts by applying the same inclusion and exclusion criteria, with the third, again, resolving disagreement. The study was finally included when all three researchers reached consensus on the inclusion and exclusion criteria.

To manage this systematic review, we used the Rayyan.ai application (13), which allowed us to import all retrieved articles, detect and discard duplicates, assign reviewers for independent and blinded screening, select relevant studies, monitor discrepancy rates, and upload and review full-text documents.

### 2.6 Data extraction

Once the studies were selected for inclusion in the review, the researchers (DPM, CMLH, and GMG), extracted the relevant data, main outcomes, and methodological quality. All collected data were entered into a Microsoft Excel spreadsheet with a template specifically designed for this purpose by the reviewers.

The extracted variables included first author, publication year, study center and recorded data, study design, sample size, percentage of nurses in the sample, type of work shift, number of participants by shift, gastrointestinal variables, assessment instruments, and positive, negative, or mixed results. These data were used to examine the general characteristics and main findings of the included studies and were subsequently compiled in Supplementary Table S3.

The methodological quality of the studies included in the review was assessed using the Newcastle–Ottawa Scale (NOS) for case-control and cohort studies (14). This scale consists of 8 items divided into 3 domains: selection, comparability, and outcome (for cohort studies) or exposure (for case-control studies). It uses a quantitative scoring system assigning one point per item, except for the comparability item, which allows up to two points, with a final score ranging from 0 to 9 points (See Supplementary Table S4).

The methodological quality of cross-sectional studies was evaluated using the Joanna Briggs Institute Critical Appraisal Tool (JBI). This tool assesses internal validity (appropriate design and conduct), reliability (consistency of results), and applicability to clinical practice (15). The appraisal criteria include sample inclusion criteria, description of study subjects and setting, valid and reliable measurement of exposure, objective measurement of the condition, identification of confounding factors, strategies to address confounding factors, valid and reliable outcome measurement, and appropriate statistical analysis. The quantitative score ranges from 0 to 8 points (See Supplementary Table S4).

### 2.7 Analysis of the meta-analysis data

Meta-analysis was conducted using odds ratios (ORs) as the effect measure, with corresponding 95% confidence intervals (CIs), to evaluate the association between shift work among nurses and the risk of gastrointestinal disorders. Both fixed-effect and random-effects models were applied based on the degree of heterogeneity among the studies.

A fixed-effect model was initially used when homogeneity was assumed. When statistical heterogeneity was indicated by Cochran's Q test and an I^2^ statistic greater than 50%, a random-effects Mantel–Haenszel meta-analysis weighted by inverse variance was applied using the DerSimonian and Laird method to account for between-study variability.

Forest plots were generated to illustrate both individual and pooled OR estimates with 95% confidence intervals. Funnel plots were used to visually assess potential publication bias. All statistical analyses were conducted using Review Manager of The Cochrane Collaboration (16). A two-tailed *p*-value of < 0.05 was considered statistically significant. Sensitivity analyses were conducted to assess the robustness of the pooled estimates. A leave-one-out procedure was performed, whereby each study was sequentially excluded and the meta-analysis repeated to determine the impact of individual studies on the overall effect size.

### 2.8 Risk of bias assessment

Risk of bias of was independently assessed by two authors using the ROBINS-E tool for non-randomized studies of exposures (17). This tool assesses the risk of bias across seven domains relevant to this type of study. These include bias due to confounding, bias arising from measurement of the exposure, bias in the selection of participants in the study and in the analysis, bias due to post-exposure interventions, bias due to missing data, bias arising from measurement of the outcome, and bias in the selection of reported results.

Studies were classified as low, moderate, or high in risk of bias according to ROBINS-E tool criteria. Those with low or moderate risk of bias were identified to provide more reliable evidence, whereas those assessed as high bias risk were interpreted with caution due to a possibility for confounding and methodological limitations.

### 2.9 Protocol and registration

This systematic review was registered in PROSPERO (International Prospective Register of Systematic Reviews) with registration number CRD420251018324. This systematic review was conducted in accordance with the Preferred Reporting Items for Systematic Reviews and Meta-Analyses (PRISMA) 2020 statement (18) and the (Supplementary material: Data Sheet 1) (19).

## 3 Results

### 3.1 Search results

**Figure 1:**
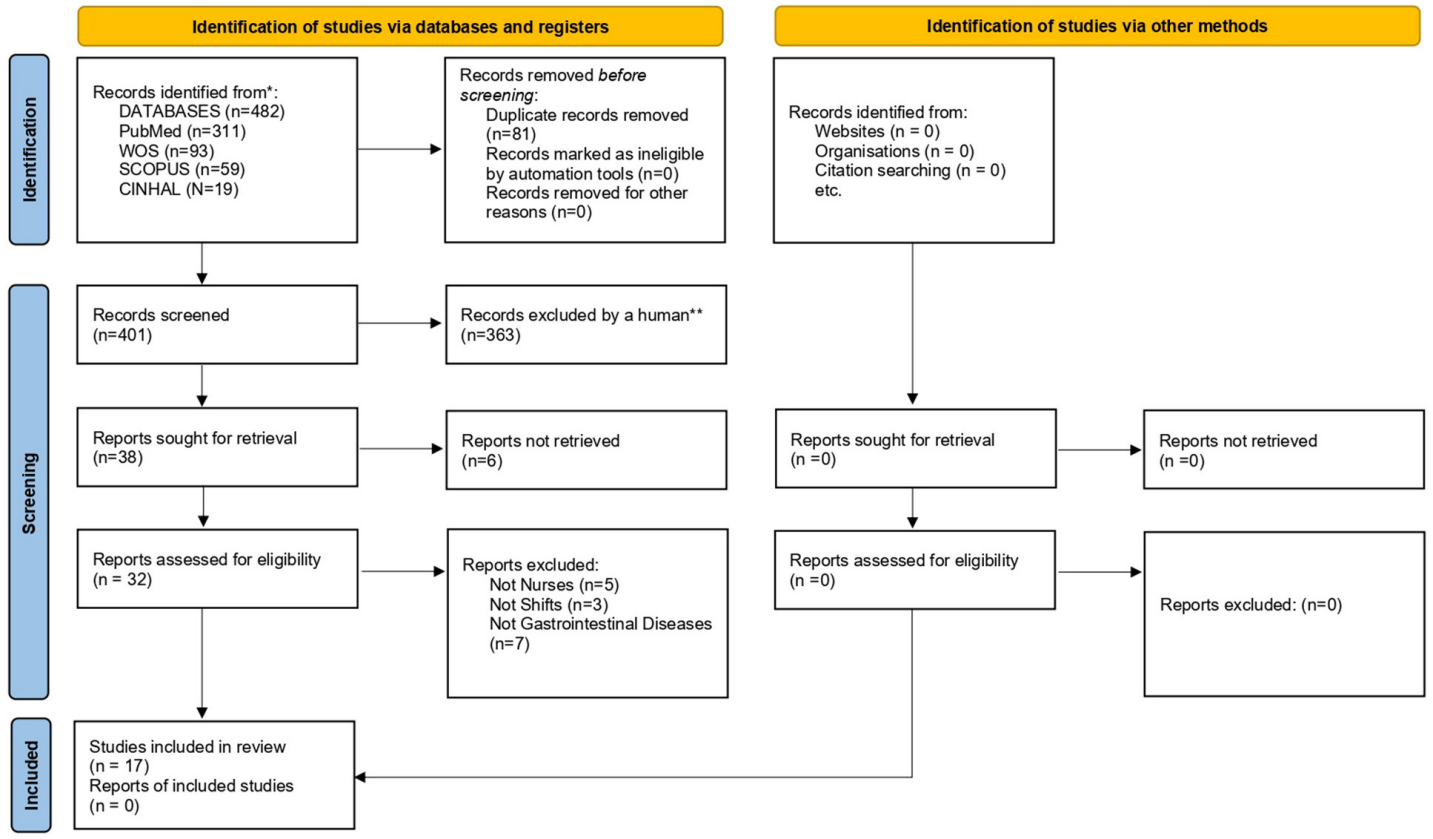
PRISMA 2020 flow diagram for new systematic reviews which included searches of databases, registers and other sources. *Consider, if feasible to do so, reporting the number of records identified from each database or register searched (rather than the total number across all databases/registers). **If automation tools were used, indicate how many records were excluded by a human and how many were excluded by automation tools. Source: Page MJ, McKenzie JE, Bossuyt PM, Boutron I, Hoffmann TC, Mulrow CD et al. The PRISMA 2020 statement: an updated guide for reporting systematic reviews. This work is licensed under a CC BY 4.0 licence. To view a copy of this licence, visit https://creativecommons.org/licenses/by/4.0/. Page MJ, et al. BMJ 2021;372:n71. doi: 10.1136/bmj.n71.

The initial database search identified 482 articles (see Figure 1), 81 were discarded as duplicates. Of the remaining 401, 363 were excluded after title and abstract screening since they did not meet inclusion criteria. This initial screening included 38 studies to be assessed by the two researchers. During this assessment, an 18% rate of discrepancies were resolved by consensus on eligibility criteria.

Thirty-two studies were selected for full-text review. Fifteen articles were excluded because their samples did not include nurses, did not report data on shift work or both; or because they did not report data on shiftwork, gastrointestinal outcomes or both. The final systematic review included 17 eligible articles with 13 studies included in the meta-analysis. Studies excluded after full-text review and reasons for exclusion are detailed in Supplementary Table S5.

### 3.2 General characteristics of the studies

Supplementary Table S3 summarizes the main characteristics and results of the selected studies. The 17 (20–36) included articles covered a data collection period from 1998 to 2023 and were published between 2003 and 2024. 35.2% (5/17) were conducted in Asia, 23.5% (4/17) in Europe, 23.5% (4/17) in the United States, and 17.8% (3/17) in other geographical regions.

The study setting was predominantly hospital-based (95.5%), followed by other healthcare institutions (4.5%), with no representation in primary care. Sample sizes ranged from a minimum of 51 to a maximum of 78,586 participants, corresponding to two cohort studies. Most samples included nurses exclusively (70.5%) or nurses combined with nursing assistants (29.5%).

All studies were of observational design, predominantly cross-sectional (70.5%, 12/17), followed by prospective longitudinal studies (17.6%, 3/17) and retrospective case-control studies (11.7%, 2/17). All studies included registered nurses in their inclusion criteria, with at least 3 years of professional experience and shift work classified as day workers (morning, afternoon, or night) or rotating shift workers (rotating day or night shifts).

For gastrointestinal disease-related variables, 82.35% of the studies collected data using self-administered or validated questionnaires (14/17), while three longitudinal studies (17.65%) used medical records to classify gastrointestinal disease (3/17). The most representative variables were those related to irritable bowel syndrome, inflammatory bowel disease, colorectal cancer, functional dyspepsia, gastrointestinal disorders, diarrhea vs. constipation, regurgitation, and gastritis vs. gastric ulcer.

### 3.3 Quality of the studies

The quality of the studies was assessed using the NOS for cohort and case-control studies and the JBI for Cross-Sectional Studies. The longitudinal studies (24, 32–34) (4/17) demonstrated higher quality than the cross-sectional studies (20–31, 35, 36) (13/17).

Among the cohort studies (24, 33, 34) (3/17), 66% (33, 34) (2/3) achieved a score of 7 out of 9 total points, and 33% (24) (1/3) achieved 6 out of 9 points. The only case-control study among the longitudinal designs (32) obtained a higher score than the previous ones, 8 out of 9. In these longitudinal studies, 100% of the study population consisted of representative community-based cohorts Nurses' Health Study (NHS) and NHS II nurses from the United States. 75% used medical records and a specific questionnaire (24, 32, 33) for the outcome variable, colorectal cancer compared with 25% (34) (1/4) for Crohn's disease and ulcerative colitis. 75% completed full follow-up on all enrolled participants (24, 32, 33). 25% achieved follow-up rates greater than 90% (34).

Among the cross-sectional studies (13/17), 30.7% (20–22, 31) scored 7 out of 8 total points; 15.3% (25, 28) scored 5 out of 8 points; 30.7% scored 4 out of 8 points (23, 29, 30, 36); and 23.07% scored 1 out of 8 points (26, 27, 35). A total of 82.3% of the studies (14/17) defined clear inclusion and exclusion criteria, with sampling predominantly based on age, workplace, years of employment, and shift type (20–25, 28, 29, 31–34, 36).

53.8% of the studies used validated questionnaires (20, 22, 23, 25, 28, 30, 31), of which the most representative was the Rome III Questionnaire for Irritable Bowel Syndrome. 61.5% of the studies minimized confounding factors through data analysis using logistic regression (20–22, 28–31, 36).

### 3.4 Effect of shift work on gastrointestinal diseases

**Figure 2:**
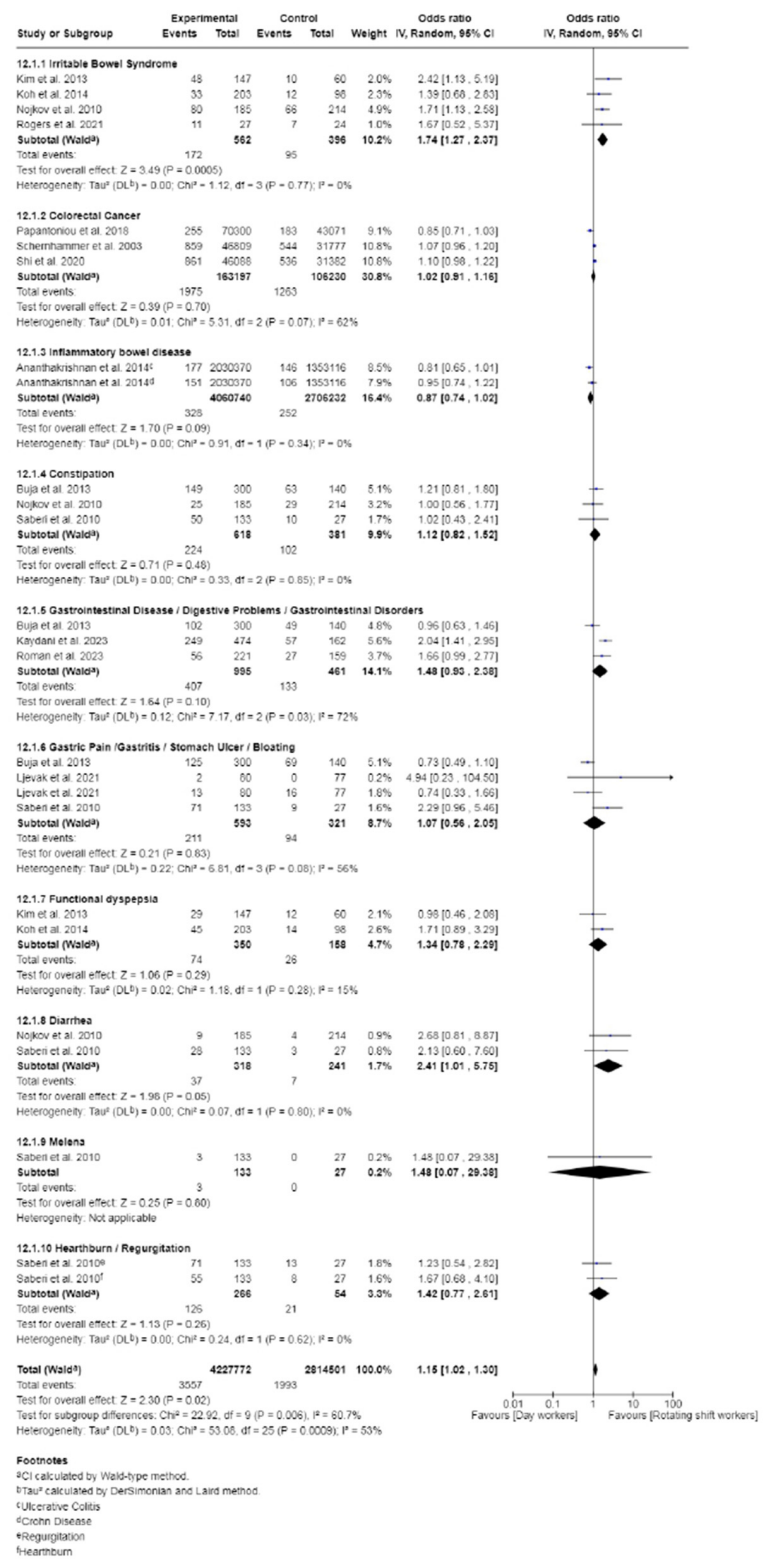
Analysis of gastrointestinal problems in Daytime vs. Rotating shifts.

The meta-analysis included 13 of the 17 studies from the systematic review. The results presented in Figure 2 indicate that rotating shifts among nurses are associated with a significant increase in the overall risk of developing gastrointestinal disorders (OR = 1.15; 95% CI: 1.02–1.30; *p* = 0.02) compared with those working day shifts. This implies that nurses working rotating shifts, including night shifts, have a 15% higher risk of developing a gastrointestinal disorder compared with nurses working exclusively day shifts.

Among the analyzed subgroups, both irritable bowel syndrome (OR = 1.74; 95% CI: 1.27–2.37) and diarrhea (OR = 2.41; 95% CI: 1.01–5.75) showed statistically significant associations, with results showing high robustness (I^2^ = 0%), finding that shift work primarily affects intestinal motility and visceral sensitivity.

No significant associations were observed with more complex organic diseases, such as colorectal cancer or inflammatory bowel disease. The moderate heterogeneity observed in the overall analysis (I^2^ = 53%, *p* < 0.05) reflects methodological and population differences across studies, although the overall effect remains consistent.

Funnel plots are shown in Supplementary Table S6.

### 3.5 Risk of bias of included studies

**Figure 3:**
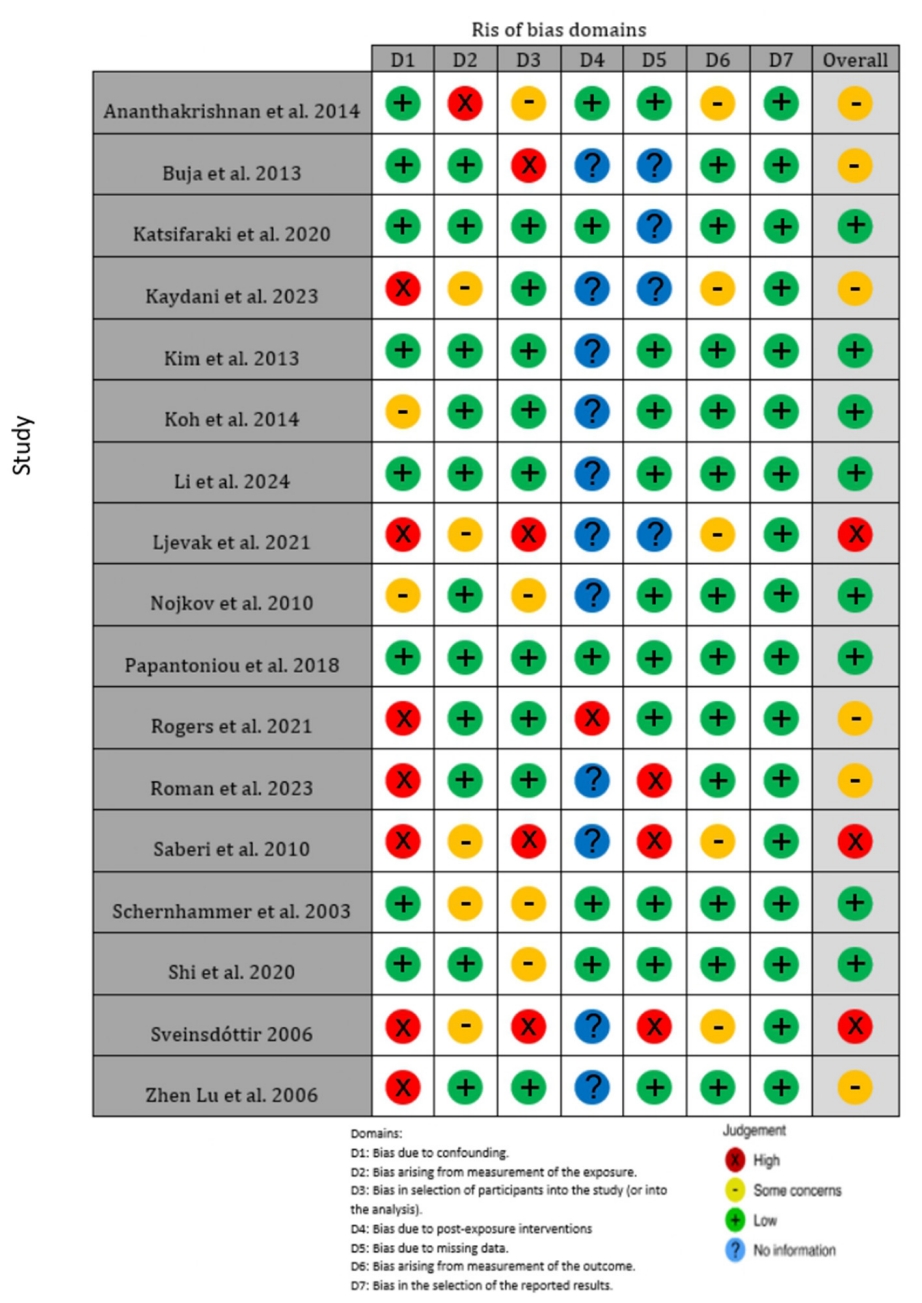
Risk of bias assessment of included studies using the ROBINS-E tool.

The ROBINS-E tool for non-randomized studies of exposures was used to assess the risk of bias in all 17 studies included in the systematic review and meta-analysis (see Figure 3). Fourteen of the 17 studies (82.35%) were classified as having a low risk of bias. Four studies showed the lowest risk of bias, with positive assessments across all domains (20, 21, 31, 33).

Three studies (26, 27, 35) were classified as having a high risk of bias due to study design. The main source of bias across the included studies was found in the selection of reported outcomes, identified in 65.4% of the studies. This was followed by bias due to confounding, present in 52.94% of the studies, and bias in the selection of study sample participants, or in the analysis in 47.06% of the studies.

## 4 Discussion

Results obtained from the meta-analyses allow the identification of consistent trends in the relationship between work shifts and the presence of gastrointestinal diseases (GID). The overall effect indicates a 15% higher probability of GID among nurses working rotating shifts, including night shifts, compared with those working day shifts exclusively.

Rotating shifts were associated with a higher risk of irritable bowel syndrome (OR = 1.74) and diarrhea (OR = 2.41), showing a strong and statistically significant association. The observed effect for IBS corresponds to the most common functional gastrointestinal disorder among nurses working rotating or night shifts (10), mainly manifested by recurrent abdominal pain, constipation, and diarrhea.

These associations may be clinically and occupationally relevant, as they are directly related to the nature of nursing competencies and the requirement for shift work in hospital settings to ensure patient care 24 hours a day, 7 days a week. This working pattern affects sleep quality, physical recovery, and cumulative exposure to irregular biomechanical loads (37). The most robust explanation is attributed to chronodisruption caused by rotating shifts, with a direct impact on nurses' gastrointestinal health (7). Between 48% and 82% of nurses working night or rotating shifts report abdominal pain, constipation, diarrhea, and other functional intestinal disorders (26, 28).

This association of signs and symptoms is shown to be independent of sleep quality suggesting a specific chronodisruption in relation to gastrointestinal pathogenesis, by modulating visceral sensitivity and altering gastrointestinal tract physiology and intestinal motility. Other frequently reported gastrointestinal disorders include diarrhea, gastric pain, dyspepsia, nausea, and loss of appetite (38, 39). Although sleep disturbance is not the sole explanatory factor for this range of gastrointestinal symptoms, it is the most important predictor associated with shift work. Among the possible mechanisms, a direct effect on the production of inflammatory cytokines that trigger symptoms has been described (40), as well as alterations in melatonin production, indicating a misalignment of the biological clock with the light–dark cycle and work schedules (41).

Other determining factors may include psychosocial factors and working conditions. Nurses working rotating shifts experience higher levels of workload and, consequently, greater stress, anxiety, and depression (42). The high prevalence of psychosocial stress among shift-working nursing staff is associated with an increased risk of gastrointestinal disorders such as abdominal pain, constipation, and diarrhea. Greater professional experience, more working hours per week, and employment in specific units such as intensive care or surgery also influence the prevalence of gastrointestinal disorders (36).

Rotating shifts that include night work make it more difficult to maintain healthy habits, such as a balanced diet based on regular schedules and more nutritious food choices. This may have a potential impact on the composition and diurnal rhythms of the intestinal microbiota, due to the overgrowth of certain bacterial groups that may impair the integrity of the intestinal barrier, increase inflammation and gastrointestinal symptoms. Furthermore, these changes in the microbiota caused by rotating shifts and their impact as a physiological stressor trigger neuroinflammatory processes that affect mental health (43).

In the relationship between gastrointestinal clinical manifestations and the study population, the main characteristic is the predominance of women in most study samples, particularly in the large NHS I and NHS II cohorts, where women make up between 85% and 97% of the total sample (34). This is consistent with other research showing more severe gastrointestinal symptoms and higher rates of irritable bowel syndrome and functional dyspepsia in women compared to men (9, 11). This may also be explained by the relatively limited representation of male nurses in study samples, highlighting the need for further research to clarify gender differences (44).

In terms of geographical context and age, the largest populations were from the United States, with a reported mean age ranging from 25 to 42 (34), followed by Europe (Spain, Italy, Bosnia and Herzegovina), with mean age between 25 and 38 (25, 27, 29) and Asia (Iran, South Korea), with reported mean ages between 28 and 37 (31, 36). The overall profile corresponds to a relatively young population, with mean professional experience ranging from 4 to 15 years.

Intensive care units, emergency departments, and inpatient hospital services are the areas in the occupational context where a greater impact of psychosocial occupational stress has been observed (45).

Study designs were predominantly cross-sectional, with an average outcome assessment period ranging from 1 to 6 months. These studies were conducted between 2002 and 2023, and the main limitations identified included lack of sample representativeness, reliance on self-reported data, and insufficient control of confounding variables. Some prospective longitudinal studies included large cohorts with mean follow-up periods of 24 years (33, 34) and 10 years (24). The consistency in exposure assessment methods and the validity of the instruments used support the overall robustness of the evidence synthesized in this review. Heterogeneity was moderate overall but low and robust for the two symptoms analyzed IBS and diarrhea with a statistical significance of *p* < 0.05.

These findings highlight the need for multicomponent prevention and intervention programs specifically targeting young female nurses aged between 25 and 45 who work rotating shifts including more than four-night shifts per month, particularly in intensive care units, emergency departments, or internal medicine services. Gastrointestinal symptoms should be reassessed and systematically monitored both in nursing professionals and in other night shift workers, given the potential long-term impact on more severe gastrointestinal pathologies such as colorectal cancer.

## 5 Conclusion

This systematic review and meta-analysis of nurses working rotating shifts demonstrates an increased risk of irritable bowel syndrome and diarrhea, with a particularly relevant impact observed in rotating schedules that include night shifts.

The observed heterogeneity, population differences and characteristics, contextualization of shift patterns, and variability in the study variables allow for an association between gastrointestinal disorders and nurses working rotating shifts.

Policy and workplace interventions should include preventive measures, early detection of cases and monitoring of gastrointestinal conditions among nurses working rotating shifts

Future, more rigorously designed research will enable a better understanding of the impact of gastrointestinal symptoms and diseases, as well as the development of targeted intervention measures to improve the health of nurses working rotating shifts.

## Data Availability

The original contributions presented in the study are included in the article/supplementary material, further inquiries can be directed to the corresponding author.

## References

[1] Meerveld G Johnson A Grundy D. Gastrointestinal physiology and function. Handb Exp Pharmacol. (2017) 239:1–16. doi: 10.1007/164_2016_118 28176047

[2] Wang Y Huang Y Chase RC Li T Ramai D Li S . Global burden of digestive diseases: a systematic analysis of the Global Burden of Diseases Study, 1990–2019. Gastroenterology. (2023) 165:773–83.e15. doi: 10.1053/j.gastro.2023.05.050 37302558

[3] Sperber AD Bangdiwala SI Drossman DA Ghoshal UC Simren M . Worldwide prevalence and burden of functional gastrointestinal disorders, results of Rome Foundation Global Study. Gastroenterology. (2021) 160:99–114.e3. doi: 10.1053/j.gastro.2020.04.014 32294476

[4] Black CJ Drossman DA Talley NJ Ruddy J Ford AC. Functional gastrointestinal disorders: advances in understanding and management. Lancet. (2020) 396:1664–74. doi: 10.1016/S0140-6736(20)32115-2 33049221

[5] McDowall K Murphy E Anderson K. The impact of shift work on sleep quality among nurses. Occup Med. (2017) 67:621–5. doi: 10.1093/occmed/kqx152 29040745

[6] Konturek PC Brzozowski T Konturek SJ. Gut clock: implication of circadian rhythms in the gastrointestinal tract. J Physiol Pharmacol. (2011) 62:139–50. 21673361

[7] Voigt RM Forsyth CB Keshavarzian A. Circadian rhythms: a regulator of gastrointestinal health and dysfunction. Expert Rev Gastroenterol Hepatol. (2019) 13:411–42. doi: 10.1080/17474124.2019.1595588 30874451 PMC6533073

[8] Yao Y You X Yang S Xie Y Yin H Shang B . Associations of long-term night shift work with incident irritable bowel syndrome: a population-based cohort study. J Gastroenterol Hepatol. (2025). 40:2231–9. doi: 10.1111/jgh.70002 40650456

[9] Hwang SK Lee YJ Cho ME Kim BK Yoon YI. Factors associated with gastrointestinal symptoms among rotating shift nurses in South Korea. Int J Environ Res Public Health. (2022) 19:9795. doi: 10.3390/ijerph19169795 36011441 PMC9408213

[10] Wang N Liu X Ye W Shi Z Bai T. Impact of shift work on irritable bowel syndrome and functional dyspepsia: a meta-analysis. Medicine. (2022) 101:e29211. doi: 10.1097/MD.0000000000029211 35758349 PMC9276432

[11] Pennaneach C Bonham MP Kelly T Gibson R Biesiekierski JR. High prevalence of disorders of irritable bowel syndrome and functional dyspepsia in night shift workers: a cross-sectional study in Australia and the United Kingdom. Clin Gastroenterol Hepatol. (2025) S1542-3565(25)01003-1. doi: 10.1016/j.cgh.2025.11.019 41308895

[12] Morgan RL Whaley P Thayer KA Schünemann HJ. Identifying the PECO: a framework for formulating good questions to explore the association of environmental and other exposures with health outcomes. Environ Int. (2018) 121:1027–31. doi: 10.1016/j.envint.2018.07.015 30166065 PMC6908441

[13] Rayyan. AI-Powered Systematic Review Management Platform [Internet]. Rayyan.ai (2025). Available online at: https://www.rayyan.ai/ (Accessed April 14, 2025).

[14] Wells GA Shea B O'Connell D Peterson J Welch V Losos M . The Newcastle–Ottawa Scale (NOS) for Assessing the Quality of Nonrandomised Studies in Meta-Analyses [Internet]. Ottawa: Ottawa Hospital Research Institute (2011).

[15] Joanna Briggs Institute. Checklist for Analytical Cross Sectional Studies [Internet]. Adelaide: JBI (2017). Available online at: https://jbi.global/critical-appraisal-tools (Accessed April 14, 2025).

[16] The Cochrane Collaboration. Review Manager (RevMan) [Computer program]. Version 5.4. Copenhagen: The Nordic Cochrane Centre, The Cochrane Collaboration (2020).

[17] Higgins JPT Morgan RL Rooney AA Taylor KW Thayer KA Silva RA . A tool to assess risk of bias in non-randomized follow-up studies of exposure effects (ROBINS-E). Environ Int. (2024) 186:108602. doi: 10.1016/j.envint.2024.108602 38555664 PMC11098530

[18] Page MJ McKenzie JE Bossuyt PM Boutron I Hoffmann TC Mulrow CD . The PRISMA 2020 statement: an updated guideline for reporting systematic reviews. BMJ. (2021) 372:n71. doi: 10.1136/bmj.n71 33782057 PMC8005924

[19] Stroup DF Berlin JA Morton SC Olkin I Williamson GD Rennie D . Meta-analysis of observational studies in epidemiology: a proposal for reporting. JAMA. (2000) 283:2008–12. doi: 10.1001/jama.283.15.2008 10789670

[20] Li J Zheng Q Jiang X Chen X Huang L Pan Y . Prevalence and bidirectional association of sleep quality and gut health among Chinese midwives: a large population, multi-center cross-sectional study. Front Public Health. (2024) 12:1368178. doi: 10.3389/fpubh.2024.1368178 38694975 PMC11061365

[21] Katsifaraki M Nilsen KB Christensen JO Wærsted M Knardahl S Bjorvatn B . Pain complaints after consecutive nights and quick returns in Norwegian nurses working three-shift rotation: an observational study. BMJ Open. (2020) 10:e035533. doi: 10.1136/bmjopen-2019-035533 PMC7482487 32912941

[22] Nojkov B Rubenstein JH Chey WD Hoogerwerf WA. The impact of rotating shift work on the prevalence of irritable bowel syndrome in nurses. Am J Gastroenterol. (2010) 105:842–7. doi: 10.1038/ajg.2010.48 20160712 PMC2887235

[23] Zhen WL Gwee KA Ho KY. Functional bowel disorders in rotating shift nurses may be related to sleep disturbances. Eur J Gastroenterol Hepatol. (2006) 18:623–7. doi: 10.1097/00042737-200606000-00008 16702851

[24] Schernhammer ES Laden F Speizer FE Willett WC Hunter DJ Kawachi I . Night-shift work and risk of colorectal cancer in the Nurses' Health Study. J Natl Cancer Inst. (2003) 95:825–8. doi: 10.1093/jnci/95.11.825 12783938

[25] Roman P Perez-Cayuela I Gil-Hernández E Rodriguez-Arrastia M Aparicio-Mota A Ropero-Padilla C . Influence of shift work on the health of nursing professionals. J Pers Med. (2023) 13:627. doi: 10.3390/jpm13040627 37109012 PMC10144026

[26] Saberi HR Moravveji AR. Gastrointestinal complaints in shift-working and day-working nurses in Iran. J Circadian Rhythms. (2010) 8:9. doi: 10.1186/1740-3391-8-9 20929565 PMC2958856

[27] Ljevak I Vasilj I Neuberg M Josipović M. The effect of shift work on the overall health status of hospital-employed nursing staff in Bosnia and Herzegovina: a cross-sectional study. Psychiatr Danub. (2021) 33:771–7. 34718317

[28] Rogers AE Hu Y Yue Y Wissel EF Petit IRA Jarrett S . Shiftwork, functional bowel symptoms, and the microbiome. PeerJ. (2021) 9:e11406. doi: 10.7717/peerj.11406 34026361 PMC8121053

[29] Buja A Zampieron A Mastrangelo G Petean M Vinelli A Cerne D . Strain and health implications of nurses' shift work. Int J Occup Med Environ Health. (2013) 26:511–21. doi: 10.2478/s13382-013-0122-2 24057261

[30] Koh SJ Kim M Oh DY Kim BG Lee KL Kim JW. Psychosocial stress in nurses with shift work schedule is associated with functional gastrointestinal disorders. J Neurogastroenterol Motil. (2014) 20:516–22. doi: 10.5056/jnm14034 25230903 PMC4204411

[31] Kim HI Jung S Choi JY Kim S Jung H Shim K . Impact of shiftwork on irritable bowel syndrome and functional dyspepsia. J Korean Med Sci. (2013) 28:431–7. doi: 10.3346/jkms.2013.28.3.431 23487413 PMC3594608

[32] Shi Y Liu L Hamada T Nowak JA Giannakis M Ma Y . Night-shift work duration and risk of colorectal cancer according to *IRS1* and *IRS2* expression. Cancer Epidemiol Biomarkers Prev. (2020) 29:133–40. doi: 10.1158/1055-9965.EPI-19-0325 31666286 PMC6954315

[33] Papantoniou K Devore EE Massa J Strohmaier S Vetter C Yang L . Rotating night shift work and colorectal cancer risk in the nurses' health studies. Int J Cancer. (2018) 143:2709–17. doi: 10.1002/ijc.31655 29978466 PMC6235706

[34] Ananthakrishnan AN Khalili H Konijeti GG Higuchi LM de SP Fuchs CS . Sleep duration affects risk for ulcerative colitis: a prospective cohort study. Clin Gastroenterol Hepatol. (2014) 12:1879–86.e1. doi: 10.1016/j.cgh.2014.04.021 24780288 PMC4209312

[35] Sveinsdóttir H. Self-assessed quality of sleep, occupational health, working environment, illness experience and job satisfaction of female nurses working different combination of shifts. Scand J Caring Sci. (2006) 20:229–37. doi: 10.1111/j.1471-6712.2006.00402.x 16756530

[36] Kaydani N Zarea K Soltanzadeh A. Gastrointestinal and cardiovascular effects of shiftwork in nurses. J Health Sci Surveill Syst. (2023) 11:48–54. doi: 10.30476/jhsss.2021.91916.1255

[37] Chang WP Peng YX. Influence of rotating shifts and fixed night shifts on sleep quality of nurses of different ages: a systematic literature review and meta-analysis. Chronobiol Int. (2021) 38:1384–96. doi: 10.1080/07420528.2021.1931273 34056959

[38] Caruso CC Lusk SL Gillespie BW. Relationship of work schedules to gastrointestinal diagnosis, symptoms, and medication use in auto factory workers. Am J Ind Med. (2004) 46:586–98. doi: 10.1002/ajim.20099 15551368

[39] Knutsson A Boggild H. Gastrointestinal disorders among shift workers. Scand J Work Environ Health. (2010) 36:85–95. doi: 10.5271/sjweh.2897 20101379

[40] Vgontzas AN Bixler EO Follett H Kales A Lin HM Zoumakis E . Adverse effects of modest sleep restriction on sleepiness, performance, and inflammatory cytokines. J Clin Endocrinol Metab. (2004) 89:2119–26. doi: 10.1210/jc.2003-031562 15126529

[41] Meyer N Harvey AG Lockley SW Dijk DJ. Circadian rhythms and disorders of the timing of sleep. Lancet. (2022) 400:1061–78. doi: 10.1016/S0140-6736(22)00877-7 36115370

[42] Chen Y Su Q Yang Y Zhang D Li Q Zhang J. Association between night shift work and mental and physical health among Chinese nurses: a cross-sectional study. Chronobiol Int. (2026) 43:72–81. doi: 10.1080/07420528.2025.2581800 41200957

[43] Mortaş H Bilici S Karakan T. The circadian disruption of night work alters gut microbiota consistent with elevated risk for future metabolic and gastrointestinal pathology. Chronobiol Int. (2020) 37:1067–81. doi: 10.1080/07420528.2020.1778717 32602753

[44] Storz MA Lombardo M Rizzo G Müller A Lederer A. Bowel health in U.S. shift workers: insights from a cross-sectional study (NHANES). Int J Environ Res Public Health. (2022) 19:3334. doi: 10.3390/ijerph19063334 35329018 PMC8954046

[45] Amiard V Telliez F Pamart F Libert JP. Health, occupational stress, and psychosocial risk factors in night shift psychiatric nurses: the influence of an unscheduled night-time nap. Int J Environ Res Public Health. (2022) 20:158. doi: 10.3390/ijerph20010158 36612478 PMC9819569

