# Supplementary material for: Impact of nursing shift patterns on work-related gastrointestinal disorders: a systematic review and meta-analysis
Source: Front Public Health. 2026 Jun 5;14:1839569. doi: 10.3389/fpubh.2026.1839569 (PMC13279410; doi:10.3389/fpubh.2026.1839569)

Supplementary Material

Supplementary Table S1. PECOS

PECOS	Inclusion	Exclusion
Population	Nurses working at the moment of the data collection	Other professional non-nurses or students
Exposure	Nurses shift work	Studies that did not included shifts<br><br>Studies focused on the COVID-19 Pandemic period
Comparator	Comparison of the presence and severity of Gastrointestinal symptoms between nurses working on rotating shifts that include night shifts versus nurses working day shifts.	Excluded if the comparison between shift work and schedule is unclear.
Outcome	Gastrointestinal disorder, e.g., irritable bowel syndrome, colorectal cancer, inflammatory bowel disease, constipation, gastric pain, functional dyspepsia, diarrhea or regurgitation,<br><br>The exposure measure through self-report questionnaires, interviews or medical history	Studies where outcome were related to other symptoms and studies that did not report results
Study design	Observational and descriptive studies, cross-sectional studies, case-control studies, or cohort studies.<br><br>Original study in full text and peer-reviewed.<br>In English and Spanish language.	Review studies (systematic reviews, meta-analyses, literature syntheses)<br><br>Intervention studies (randomized clinical trials)<br><br>Protocol studies

	No time limits.	Theses, dissertations, or conference presentations

Supplementary Table S2. Literature Research

Block Nurse	
"Nurse	(nurse) OR ("Nurses"[MeSH Terms]) OR (“Nursing"[MeSH Terms]) OR ("Nursing Staff"[MeSH Terms]) OR (Nursing Personnel)
"Nurses"[MeSH Terms]	
“Nursing"[MeSH Terms]	
"Nursing Staff"[MeSH Terms]	
"Nursing Personnel"	
Block Shift	
"Shift Work Schedule"[MeSH Terms]	(rotating shift) OR (fixed night shift) OR (fixed day shift) OR (irregular shift) OR (shiftwork) OR ("Shift Work Schedule"[MeSH Terms]) OR (Night Shift Work) OR (rotating shift work) OR ("Work Schedule Tolerance"[MeSH Terms])
"Work Schedule Tolerance"[MeSH Terms]	
"Rotating shift"	
"Fixed night shift"	
"Fixed day shift"	
"Irregular shift"	
"Shiftwork"	
"Night Shift Work"	
Block Gastrointestinal	

"Gastrointestinal diseases"[MeSH Terms]	("Gastrointestinal diseases"[MeSH Terms]) OR ("Signs and symptoms"[MeSH Terms]) OR ("Upper Gastrointestinal Tract"[MeSH Terms]) OR ("Lower Gastrointestinal Tract"[MeSH Terms]) OR (bowel disorders) OR (gastrointestinal symptoms) OR (gastrointestinal disorders) OR (stomach problems) OR (stomach disorders) OR (digestive problems) OR (digestive symptoms) OR (intestinal problems) OR (gastrointestinal disturbances) OR (digestive troubles) OR (gastrointestinal health issues)
"Signs and symptoms"[MeSH Terms]	
"Upper Gastrointestinal Tract"[MeSH Terms]	
"Lower Gastrointestinal Tract"[MeSH Terms]	
"Bowel disorders"	
"Gastrointestinal symptoms"	
"Gastrointestinal disorders"	
"Stomach problems"	
"Stomach disorders"	
"Digestive problems"	
"Digestive symptoms"	
"Intestinal problems"	
"Gastrointestinal disturbances"	
"Digestive troubles"	
"Gastrointestinal health issues"	

Search strategy:

(((nurse) OR ("Nurses"[MeSH Terms]) OR ("Nursing"[MeSH Terms]) OR ("Nursing Staff"[MeSH Terms]) OR (Nursing Personnel)) AND ((rotating shift) OR (fixed night shift) OR (fixed day shift) OR (irregular shift) OR (shiftwork) OR ("Shift Work Schedule"[MeSH Terms]) OR (Night Shift Work) OR (rotating shift work) OR ("Work Schedule Tolerance"[MeSH Terms]))) AND ((("Gastrointestinal diseases"[MeSH Terms]) OR ("Signs and symptoms"[MeSH Terms]) OR ("Upper Gastrointestinal Tract"[MeSH Terms]) OR ("Lower Gastrointestinal Tract"[MeSH Terms]) OR (bowel disorders) OR

(gastrointestinal symptoms) OR (gastrointestinal disorders) OR (stomach problems) OR (stomach disorders) OR (digestive problems) OR (digestive symptoms) OR (intestinal problems) OR (gastrointestinal disturbances) OR (digestive troubles) OR (gastrointestinal health issues))

Supplementary Table S3. General characteristics of the studies

Author / Date / Country	Year Data Collection	Center (Unicenter / Multicenter)	Mean Age	Study	Total Sample Size	% of nurses	Shifts	Shifts (N)	Gastrointestinal Variables	Measurement Tools	Results
Li, et al / 2024 / China	2023	Multicenter	All ages	Cross-sectional	2041	100%	Rotating<br><br>No-	1636	Intestinal Health (intestinal status, dolor abdominal, estreñimiento, eating habits and defecation)	Gut-brain health questionaire (GBHQ) (high score worse gut health)	+
							Rotating	405			
Katsifari, et al / 2023 / Norway	2014-2015	Multicenter	18-63 years	Cross-sectional	679	100%	Morning	309	Abdominal Pain	Dairy queationnaire (Likert-type scale from 0 to 3)	+
							Afternoon	162			
							Night	278			
Nojkov, et al / 2010 / USA	2007-08	Multicenter	45.6 ± 9.3 years (fixed day)<br><br>41.5 ± 11 years (fixed night)<br><br>37.3 ± 10.8 years (rotating)	Cross-sectional	399	100%	Fixed day	214	Irritable Bowel Syndrome	Rome III Questionnarie	+
							Fixed night	110	Irritable Bowel Syndrome		-

									constipation-predominant	IBS-QOL	
							Rotating	75			
									Irritable Bowel Syndrome diarrhea-predominant		/
									Mixed Irritable Bowel Syndrome		+
									Functional constipation		-
									Functional diarrhea		+
									Abdominal pain		+
Zhen Lu, et al / 2006 / Singapore	2005	Unicenter	Range 20-60 years<br>Median 29 years	Cross-sectional	118	100%	Rotating shift nurses	58	Functional bowel disorders (FBD)		+
									Dyspectic Symptom		/
							Regular Day shifts	60			

										Gastrointestinal Symptom Questionnaire<br><br>IBS Symptoms Evaluation Questionnaire	
Schernhammer, et al / 2003 / USA	1988-1988	Multicenter registry NHS I cohort USA	54.3 – 57.1 years	Longitudinal cohort	78586	100%	Rotating (1-14 years)	31777	Colon Cancer	Autoinformes and Medical history	/
							Rotating (≥15 years)	40990	Rectal Cancer		+<br>(≥15 years)
							Non-rotating	5819			
Roman, et al / 2023 / Spain	2019	Multicenter	24.8 years (fixed shift)<br><br>28 years (rotating shift)	Cross-sectional	380	100%	Rotating Shift	221	Digestive symptoms (reflux, diarrhea, constipation, abdominal pain and	Gastrointestinal Symptom Rating Scale (GSRS global Score)	+<br><br>(abdominal pain, indigestion

							Fixed-Shift	159	indigestion)		
Saberi, et al / 2010 / Iran	2008-09	Unicenter	35 years (Day Shifts)<br><br>38.5 years (Rotating)	Cross-sectional	160	100%	Day shifts	27	Gastrointestinal symptoms (heartburn, regurgitation, constipation, diarrhea and bloating)	GI symptom Questionnaire	+
							Rotating	113			
Ljevak, et al / 2021 / Bosnia Herzegobina	2019	Unicenter	33.3 years	Cross-sectional comparative	157	100%	Day shifts	77	Appetite loss, nausea, heartburn	Standard Shiftwork Index (SSI)	+
							Rotating (12h day shift/24h off and 12h night shift/48h off)	80			
Rogers, et al / 2021 / USA	N/A	Unicenter	32.4 years (day shift) – 33.3 years (night shift)	Comparative observational	51	100%	Day shifts	24	Irritable bowel syndrome (abdominal pain, bloating, diarrhea, constipation),<br><br>Microbiotal diversity and abundance	Rome III Questionnaire<br><br>Fecal (ARNr)	/
							Nigh shift (rotating)	27			
Buja, et al / 2013 / Italy	2011	Unicenter	42.0 ± 6.4 years (no shifts)	Cross-sectional	440	100%	Day without nights	94	Gastrointestinal Symptoms (constipation, digestive problems, gastric pain)		+<br><br>(gastric pain)

			42.8 ± 6.7 years (no night shifts)<br><br>35.9 ± 8.1 years (night shifts)				Rotating nights	300		Job Content Questionnaire (JCQ)	
							Morning shift	46			
Koh, et al / 2014 / Korea	2012-13	Unicenter	28.12 ± 4.88 years (subjects with Functional Gastrointestinal Disorders)<br><br>29.48 ± 4.88 6.43 years (subjects without Functional Gastrointestinal Disorders)	Cross-sectional	301	48%	Rotating shift workers	203	Irritable Bowel Syndrome (IBS)	Rome III Questionnaire<br><br>Korean versión of Bowel Disease Questionnaire	+
							Day shift workers	98	Constipation-predominant Irritable Bowel Syndrome		
									Diarrhea-predominant Irritable Bowel Syndrome		
									Functional Dyspepsia		
									Mixed Irritable Bowel Syndrome		
									Unsubtyped Irritable Bowel Syndrome		

Kim, et al / 2013 / Korea	2010-11	Unicenter	31.43 ± 8.20 years (rotating workers)<br><br>34.30 ± 6.43 years (day workers)	Cross-sectional	207	82%	Rotating workers	147	Irritable Bowel Syndrome	Rome III Questionnaire	+
							Day workers	60	Functional Dyspepsia	Korean Bowel disease questionnaires (K-BDQ)	+
Shi, et al / 2020 / USA	1988-2012	Multicenter Registry NHS USA	NHS I cohort: 30–55 years<br><br>NHS II cohort: 25–42 years	Case-control	1397	100%	Night	536	Colorectal Cancer	MEDICAL RECORD or REPORTS of Colorectal Cancer with biennial questionnaire: confirmed cases, possible cases, anatomical location, stage, histological type	+
							1–14 years with shifts including night	718			
							15 years with shifts including night	143			
Papantoniou, et al / 2018 / USA	1988-2013	Multicenter Registry NHS USA	NHS I cohort: 30–55 years	Prospective cohorts	NHS 1 (77349)	100%	Rotating	77349	Colon Cancer CRC (epithelial, carcinoid, non-epithelial) and	MEDICAL RECORD or REPORTS of Colorectal	

			NHS II cohort: 25–42 years		NHS 2 (113371)		Non Rotating	113371	subsites (proximal, distal or rectum))	Cancer with biennial questionnaire: confirmed cases, possible cases, anatomical location, stage, histological type	/
Ananthakrishnan, et al / 2014 / USA	1986 (NHS I)-2001 (NHS II)	Multicenter registry NHS USA	NHS I cohort: 30–55 years<br><br>NHS II cohort: 25–42 years	Prospective cohorts	151,871	100%	Rotating shifts (at least 3 nights/month)<br><br>1-5 years	1,516,221 person/year	Crohn's Disease:<br><br>Ulcerative Colitis:	Medical Record or Reports	/
							Rotating shifts (at least 3 nights/month)<br><br>≥6 years	514,149 person/year	Crohn's Disease:<br><br>Ulcerative Colitis:		
							Never	1.353.116 person/year	Crohn's Disease:<br><br>Ulcerative Colitis:		
Sveinsdottir, et al / 2006 / Iceand	2002	Multicenter Registry Icelandic Nurses Association	45.8 ± 8.1years (Day-morning)<br><br>45.5 ± 9.2 years (Day-Evening)	Cross-sectional	348	100%	Day (Morning)	154	2-symptom gastrointestinal clinical scale	Self-administered questionnaire on occupational health, sleep quality, occupational	+

			40.0 ± 9.3years (Day-Evening-Night)				Day-Evening	85		diseases, job satisfaction and work environment (Women´s Health)	(Day-Evening)
							Day/evening/night	95			
Kaydani, et al/ 2023 / Iran	2020	Multicenter	37.6 ± 5.3 years (day working)<br><br>36.9 ± 5.14 years (Shift-working)	Cross-sectional	636	100%	Day shift	162	Gastrointestinal disorders (increased or decreased appetite, constipation/diarrhea, indigestion, gastric ulcers and stomach pain/heartburn)	Comprehensive questionnaire designed and developed by Choobineh, et al	+
							Rotating	474			

+: the study results shows positive and significative association; -* the results shows not show association: +/-: the results show an association and significance for some variables and not for others.

Supplementary Table S14. NOS and JBI

**ARTICLES**			
**COHORT STUDIES**	**Schernhammer, et al. (2003)**	**Papantoniou, et al. (2018)**	**Ananthakrishnan, et al. (2014)**
**SELECTION**			
1) Representativeness of the exposed cohort			
a) Truly representative of the average _______________ (describe) of the community *	NHS 1 Cohort USA	NHS 1 and NHS2 Cohort USA	NHS 1 and NHS2 Cohort USA
b) Somewhat representative of the average ______________ of the community *			
c) Selected group of users, e.g., nurses, volunteers			
d) Without description of the cohort's origin			
2) Selection of the unexposed cohort			
a) From the same community as the exposed cohort *	*	*	*
b) Coming from a different source.			
c) No description of the origin of the unexposed cohort.			
3) Verification of exposure.			
a) Secure record (e.g., surgical records). *		*	*

b) Structured interview. *			
c) Report written by the interested party themselves.			
d) No description.	X		
4) Demonstration that the outcome of interest was not present at the start of the study.			
a) Yes. *	*	*	*
b) No.			
**COMPARABILITY ****			
1) Comparability of cohorts based on design or analysis.			
a) The study controls for _____________ (select the most important factor). *	Colorectal cancer and nurses' health	Colorectal cancer and specific subsites	Crohn's Disease and Ulcerative Colitis
b) The study controls for any additional factors. ¯ (This criterion could be modified to indicate specific control			
of a second important factor).			
**RESULT**			
1) Evaluation of the result			
a) Independent blind evaluation *			
b) Record linkage *			
c) Self-report	Questionnaire	Biennial self-reported questionnaire with	Biennial self-reported questionnaire with self-

		access to medical records	reported diagnosis
d) No description			
2) Was the follow-up long enough for the results to occur?			
a) Yes (select an appropriate follow-up period for the outcome of interest) *	* (1988-1998)	* (1988-2012 and 1989-2013)	* (1988-2012 and 1989-2013)
b) No			
3) Adequacy of cohort follow-up			
a) Complete follow-up: all subjects counted *	*	*	
b) Subjects lost during follow-up are unlikely to introduce bias: small number of losses: > ____% (select an appropriate %) of follow-up, or description provided of those lost) *			Tracking greater than 90%
c) Follow-up rate < ____% (select an appropriate %) and no description of those lost			
d) No declaration			
**TOTAL SCORE**	**6 of 9**	**7 of 9**	**7 of 9**

**ARTICLES**	
**COHORT STUDIES**	**Shi, et al. (2020)**
**SELECTION**	
1) Representativeness of the exposed cohort	

a) Truly representative of the average _______________ (describe) of the community *	Medical records
b) Somewhat representative of the average ______________ of the community *	
c) Selected group of users, e.g., nurses, volunteers	
d) Without description of the cohort's origin	
2) Selection of the unexposed cohort	NHS 1 Cohort USA
a) From the same community as the exposed cohort *	
b) Coming from a different source.	
c) No description of the origin of the unexposed cohort.	Same disease-free cohort
3) Verification of exposure.	
a) Secure record (e.g., surgical records). *	
b) Structured interview. *	
c) Report written by the interested party themselves.	Disease-free
d) No description.	
4) Demonstration that the outcome of interest was not present at the start of the study.	Medical records
a) Yes. *	
b) No.	

**COMPARABILITY ****	
1) Comparability of cohorts based on design or analysis.	Colorectal cancer
a) The study controls for _____________ (select the most important factor). *	
b) The study controls for any additional factors. ¯ (This criterion could be modified to indicate specific control	
of a second important factor).	
**RESULT**	
1) Evaluation of the result	* biennial medical records and pathology reports
a) Independent blind evaluation *	
b) Record linkage *	
c) Self-report	
d) No description	
2) Was the follow-up long enough for the results to occur?	
a) Yes (select an appropriate follow-up period for the outcome of interest) *	*
b) No	
3) Adequacy of cohort follow-up	
a) Complete follow-up: all subjects counted *	* Only due to lack of records
b) Subjects lost during follow-up are unlikely to introduce bias: small number of losses: > ____% (select an appropriate %)	

of follow-up, or description provided of those lost) *	
c) Follow-up rate < ____% (select an appropriate %) and no description of those lost	
**TOTAL SCORE**	**8 of 9**

**ARTICLES**				
**Cross-sectional studies**	**Li, et al. (2024)**	**Katsifari, et al. (2020)**	**Nojkow, et al. (2010)**	**Zhen Lu, et al. (2006)**
Were the inclusion criteria for the sample clearly defined?				
The authors should provide clear inclusion and exclusion criteria, developed prior to the recruitment of study participants. These criteria should be specified (e.g., risk, stage of disease progression) in sufficient detail, along with all essential information for the study.	yes	yes	yes	yes
Were the study subjects and the setting described in detail?				
The study sample should be described in enough detail for other researchers to determine whether it is comparable to the population of interest. The authors should provide a clear description of the population from which the study participants were selected or recruited, including demographic data, location, and time period.	yes	yes	no	yes
Was the exposure measured in a valid and reliable way?				
The study should clearly describe the method used to measure the exposure. Assessing validity requires the availability of a reference standard with which the measurement can be compared. The validity of exposure measurement is usually related to the relevance of a current measure or the need for a measure of past exposure. Reliability refers to the processes included in an epidemiological study to check the repeatability of exposure measurements. These usually include intra-observer and inter-observer reliability.	yes	yes	yes	yes

Were objective, standardised criteria used for the measurement of the condition?				
It is helpful to determine whether patients were included in the study on the basis of a specific diagnosis or definition. This is likely to reduce the risk of bias. Characteristics are another useful approach for group matching, and studies that did not use specific diagnostic methods or definitions should provide evidence of matching on key characteristics.	No	no	No	no
Were confounding factors identified?				
Confounding occurs when the estimated effect of exposure to an intervention is biased by the presence of some difference between the comparison groups (other than the exposure being investigated/of interest). Typical confounders include baseline characteristics, prognostic factors, or concomitant exposures (e.g., smoking). A confounder is a difference between the comparison groups that influences the direction of the study outcomes. A high-quality cohort study design will identify possible confounders and measure them (where possible). This is difficult in studies where behavioural, attitudinal, or lifestyle factors may influence outcomes.	Yes	Yes	it is not clear	no
Were strategies to deal with confounding factors stated?				
Strategies to address the effects of confounders may be implemented in the study design or in the data analysis. Through matching or stratifying the sampling of participants, the effects of confounding factors can be adjusted. When adjustment is carried out during data analysis, the reviewer should assess the statistical methods used in the study. Most will involve some form of multivariate regression analysis to account for the measured confounders.	Yes	Yes	it is not clear	yes
Were the outcomes measured in a valid and reliable way?				
Read the methods section of the article. If, for example, lung cancer is assessed on the basis of existing definitions or diagnostic criteria, the answer to this question is likely to be yes. If lung cancer is evaluated using observer-reported or self-reported scales, the risk of over-reporting or under-reporting increases, compromising objectivity. It is important to determine whether the measurement tools used were validated instruments, as this has a significant impact on the validity of outcome assessment. Once the objectivity of the outcome measurement instrument (e.g., lung cancer) is established, it is important to establish how the measurement was conducted. Were those involved in data collection (e.g., radiologists) trained in the use of the instruments? If more than one	yes	yes	yes	no

data collector was involved, were they similar in terms of educational level, clinical or research experience, or level of responsibility in the study being assessed?				
Was appropriate statistical analysis used?				
As with any consideration of statistical analysis, it is important to consider whether there was a more appropriate alternative statistical method that could have been used. The methods section should be sufficiently detailed for reviewers to identify which analytical techniques were used (in particular, regression or stratification) and how specific confounders were measured. For studies utilising regression analysis, it is useful to determine whether the study identified which variables were included and how they related to the outcome. If stratification was used as the analytical approach, were the strata of analysis defined by the specified variables? Additionally, it is important to assess the appropriateness of the analytical strategy in relation to the assumptions associated with the approach, as different analytical methods are based on different assumptions about the data and its behaviour.	yes	yes	yes	no

**ARTICLES**				
**Cross-sectional studies**	**Roman et al. (2023)**	**Saberi, et al. (2010)**	**Ljevak, et al. (2021)**	**Rogers, et al. (2021)**
Were the inclusion criteria for the sample clearly defined?				
The authors should provide clear inclusion and exclusion criteria, developed prior to the recruitment of study participants. These criteria should be specified (e.g., risk, stage of disease progression) in sufficient detail, along with all essential information for the study.	yes	no	no	yes
Were the study subjects and the setting described in detail?				

The study sample should be described in enough detail for other researchers to determine whether it is comparable to the population of interest. The authors should provide a clear description of the population from which the study participants were selected or recruited, including demographic data, location, and time period.	yes	yes	no	yes
Was the exposure measured in a valid and reliable way?				
The study should clearly describe the method used to measure the exposure. Assessing validity requires the availability of a reference standard with which the measurement can be compared. The validity of exposure measurement is usually related to the relevance of a current measure or the need for a measure of past exposure. Reliability refers to the processes included in an epidemiological study to check the repeatability of exposure measurements. These usually include intra-observer and inter-observer reliability.	yes	it is not clear	it is not clear	yes
Were objective, standardised criteria used for the measurement of the condition?				
It is helpful to determine whether patients were included in the study on the basis of a specific diagnosis or definition. This is likely to reduce the risk of bias. Characteristics are another useful approach for group matching, and studies that did not use specific diagnostic methods or definitions should provide evidence of matching on key characteristics.	No	No	no	yes
Were confounding factors identified?				
Confounding occurs when the estimated effect of exposure to an intervention is biased by the presence of some difference between the comparison groups (other than the exposure being investigated/of interest). Typical confounders include baseline characteristics, prognostic factors, or concomitant exposures (e.g., smoking). A confounder is a difference between the comparison groups that influences the direction of the study outcomes. A high-quality cohort study design will identify possible confounders and measure them (where possible). This is difficult in studies where behavioural, attitudinal, or lifestyle factors may influence outcomes.	yes	no	yes	it is not clear
Were strategies to deal with confounding factors stated?				

Strategies to address the effects of confounders may be implemented in the study design or in the data analysis. Through matching or stratifying the sampling of participants, the effects of confounding factors can be adjusted. When adjustment is carried out during data analysis, the reviewer should assess the statistical methods used in the study. Most will involve some form of multivariate regression analysis to account for the measured confounders.	No	No	No	yes
Were the outcomes measured in a valid and reliable way?				
Read the methods section of the article. If, for example, lung cancer is assessed on the basis of existing definitions or diagnostic criteria, the answer to this question is likely to be yes. If lung cancer is evaluated using observer-reported or self-reported scales, the risk of over-reporting or under-reporting increases, compromising objectivity. It is important to determine whether the measurement tools used were validated instruments, as this has a significant impact on the validity of outcome assessment. Once the objectivity of the outcome measurement instrument (e.g., lung cancer) is established, it is important to establish how the measurement was conducted. Were those involved in data collection (e.g., radiologists) trained in the use of the instruments? If more than one data collector was involved, were they similar in terms of educational level, clinical or research experience, or level of responsibility in the study being assessed?	yes	it is not clear	it is not clear	no
Was appropriate statistical analysis used?				
As with any consideration of statistical analysis, it is important to consider whether there was a more appropriate alternative statistical method that could have been used. The methods section should be sufficiently detailed for reviewers to identify which analytical techniques were used (in particular, regression or stratification) and how specific confounders were measured. For studies utilising regression analysis, it is useful to determine whether the study identified which variables were included and how they related to the outcome. If stratification was used as the analytical approach, were the strata of analysis defined by the specified variables? Additionally, it is important to assess the appropriateness of the analytical strategy in relation to the assumptions associated with the approach, as different analytical methods are based on different assumptions about the data and its behaviour.	No	No	No	no

ARTICLES				
**Cross-sectional studies**	**Buja, et al. (2013)**	**Koh, et al. (2014)**	**Kim, et al. (2013)**	**Sveinsdóttir, et al. (2006)**
Were the inclusion criteria for the sample clearly defined?				
The authors should provide clear inclusion and exclusion criteria, developed prior to the recruitment of study participants. These criteria should be specified (e.g., risk, stage of disease progression) in sufficient detail, along with all essential information for the study.	yes	no	yes	it is not clear
Were the study subjects and the setting described in detail?				
The study sample should be described in enough detail for other researchers to determine whether it is comparable to the population of interest. The authors should provide a clear description of the population from which the study participants were selected or recruited, including demographic data, location, and time period.	yes	no	yes	yes
Was the exposure measured in a valid and reliable way?				
The study should clearly describe the method used to measure the exposure. Assessing validity requires the availability of a reference standard with which the measurement can be compared. The validity of exposure measurement is usually related to the relevance of a current measure or the need for a measure of past exposure. Reliability refers to the processes included in an epidemiological study to check the repeatability of exposure measurements. These usually include intra-observer and inter-observer reliability.	it is not clear	Yes	yes	no
Were objective, standardised criteria used for the measurement of the condition?				
It is helpful to determine whether patients were included in the study on the basis of a specific diagnosis or definition. This is likely to reduce the risk of bias. Characteristics are another useful approach for group matching, and studies that did not use specific diagnostic methods or definitions should provide evidence of matching on key characteristics.	No	No	No	no

Were confounding factors identified?				
Confounding occurs when the estimated effect of exposure to an intervention is biased by the presence of some difference between the comparison groups (other than the exposure being investigated/of interest). Typical confounders include baseline characteristics, prognostic factors, or concomitant exposures (e.g., smoking). A confounder is a difference between the comparison groups that influences the direction of the study outcomes. A high-quality cohort study design will identify possible confounders and measure them (where possible). This is difficult in studies where behavioural, attitudinal, or lifestyle factors may influence outcomes.	No	it is not clear	Si	no
Were strategies to deal with confounding factors stated?				
Strategies to address the effects of confounders may be implemented in the study design or in the data analysis. Through matching or stratifying the sampling of participants, the effects of confounding factors can be adjusted. When adjustment is carried out during data analysis, the reviewer should assess the statistical methods used in the study. Most will involve some form of multivariate regression analysis to account for the measured confounders.	yes	yes	Yes	no
Were the outcomes measured in a valid and reliable way?				
Read the methods section of the article. If, for example, lung cancer is assessed on the basis of existing definitions or diagnostic criteria, the answer to this question is likely to be yes. If lung cancer is evaluated using observer-reported or self-reported scales, the risk of over-reporting or under-reporting increases, compromising objectivity. It is important to determine whether the measurement tools used were validated instruments, as this has a significant impact on the validity of outcome assessment. Once the objectivity of the outcome measurement instrument (e.g., lung cancer) is established, it is important to establish how the measurement was conducted. Were those involved in data collection (e.g., radiologists) trained in the use of the instruments? If more than one data collector was involved, were they similar in terms of educational level, clinical or research experience, or level of responsibility in the study being assessed?	it is not clear	Yes	Yes	no

Was appropriate statistical analysis used?				
As with any consideration of statistical analysis, it is important to consider whether there was a more appropriate alternative statistical method that could have been used. The methods section should be sufficiently detailed for reviewers to identify which analytical techniques were used (in particular, regression or stratification) and how specific confounders were measured. For studies utilising regression analysis, it is useful to determine whether the study identified which variables were included and how they related to the outcome. If stratification was used as the analytical approach, were the strata of analysis defined by the specified variables? Additionally, it is important to assess the appropriateness of the analytical strategy in relation to the assumptions associated with the approach, as different analytical methods are based on different assumptions about the data and its behaviour.	yes	yes	yes	yes

**ARTICLES**	
**Cross-sectional studies**	**Kaydani, et al. (2023)**
Were the inclusion criteria for the sample clearly defined?	
The authors should provide clear inclusion and exclusion criteria, developed prior to the recruitment of study participants. These criteria should be specified (e.g., risk, stage of disease progression) in sufficient detail, along with all essential information for the study.	yes
Were the study subjects and the setting described in detail?	
The study sample should be described in enough detail for other researchers to determine whether it is comparable to the population of interest. The authors should provide a clear description of the population from which the study participants were selected or recruited, including demographic data, location, and time period.	yes
Was the exposure measured in a valid and reliable way?	

The study should clearly describe the method used to measure the exposure. Assessing validity requires the availability of a reference standard with which the measurement can be compared. The validity of exposure measurement is usually related to the relevance of a current measure or the need for a measure of past exposure. Reliability refers to the processes included in an epidemiological study to check the repeatability of exposure measurements. These usually include intra-observer and inter-observer reliability.	no
Were objective, standardised criteria used for the measurement of the condition?	
It is helpful to determine whether patients were included in the study on the basis of a specific diagnosis or definition. This is likely to reduce the risk of bias. Characteristics are another useful approach for group matching, and studies that did not use specific diagnostic methods or definitions should provide evidence of matching on key characteristics.	it is not clear
Were confounding factors identified?	
Confounding occurs when the estimated effect of exposure to an intervention is biased by the presence of some difference between the comparison groups (other than the exposure being investigated/of interest). Typical confounders include baseline characteristics, prognostic factors, or concomitant exposures (e.g., smoking). A confounder is a difference between the comparison groups that influences the direction of the study outcomes. A high-quality cohort study design will identify possible confounders and measure them (where possible). This is difficult in studies where behavioural, attitudinal, or lifestyle factors may influence outcomes.	no
Were strategies to deal with confounding factors stated?	
Strategies to address the effects of confounders may be implemented in the study design or in the data analysis. Through matching or stratifying the sampling of participants, the effects of confounding factors can be adjusted. When adjustment is carried out during data analysis, the reviewer should assess the statistical methods used in the study. Most will involve some form	yes

of multivariate regression analysis to account for the measured confounders.	
Were the outcomes measured in a valid and reliable way?	
Read the methods section of the article. If, for example, lung cancer is assessed on the basis of existing definitions or diagnostic criteria, the answer to this question is likely to be yes. If lung cancer is evaluated using observer-reported or self-reported scales, the risk of over-reporting or under-reporting increases, compromising objectivity. It is important to determine whether the measurement tools used were validated instruments, as this has a significant impact on the validity of outcome assessment. Once the objectivity of the outcome measurement instrument (e.g., lung cancer) is established, it is important to establish how the measurement was conducted. Were those involved in data collection (e.g., radiologists) trained in the use of the instruments? If more than one data collector was involved, were they similar in terms of educational level, clinical or research experience, or level of responsibility in the study being assessed?	it is not clear
Was appropriate statistical analysis used?	
As with any consideration of statistical analysis, it is important to consider whether there was a more appropriate alternative statistical method that could have been used. The methods section should be sufficiently detailed for reviewers to identify which analytical techniques were used (in particular, regression or stratification) and how specific confounders were measured. For studies utilising regression analysis, it is useful to determine whether the study identified which variables were included and how they related to the outcome. If stratification was used as the analytical approach, were the strata of analysis defined by the specified variables? Additionally, it is important to assess the appropriateness of the analytical strategy in relation to the assumptions associated with the approach, as different analytical methods are based on different assumptions about the data and its behaviour.	yes

Supplementary Table S5. Studies and reasons not included in the systematic review

Year, Author	Title	Reason excluded
2012, Vasconcelos S	Morbidity among nursing personnel and its association with working conditions and work organization.	No Gastrointestinal disorders
2017, Kim J	A Comparison of Standard Shiftwork Index between Night Shift Fixed Nurses and Rotating Shift Nurses	No Gastrointestinal disorders
2020, Soltaninejad M	Shift working disorders among nurses of Tehran hospital and its related factors in 2016	No association between Gastrointestinal disorders and sifhts reported
2023, Amiard V	Health, Occupational Stress, and Psychosocial Risk Factors in Night Shift Psychiatric Nurses: The Influence of an Unscheduled Night-Time Nap	No Gastrointestinal disorders
2008, Wang X	[Investigation of functional dyspepsia and functional gastrointestinal diseases in shift nurses	No association between gastrointestinal disorders and sifhts reported
2019, Katsifaraki, M	Sleep duration mediates abdominal and lower-extremity pain after night work in nurses	No sample nurses
2023, Nagarethinam M	Functional gastrointestinal disorders among healthcare professionals at a tertiary Australian hospital	No Gastrointestinal disorders
2021, De Rijk, M	The association between eating frequency with alertness and gastrointestinal complaints in nurses during the night shift	No Gastrointestinal disorders
2006, Bilski B	Influence of shift work on the diet and gastrointestinal complains among nurses. A pilot study	Foreign language: polaco
2022, Waage S	Irritabel bowel disease among norwegian nurses - associations with insomnia, excessive sleepiness, shift work disorder and shift schedule	No association between gastrointestinal disorders and sifhts reported

1978, Estryn B	Effects of night shift working upon a feminine population. Results of a survey in the hospital sector	No sample nurses
2017, Khammar, A	Survey of shift work disorders and occupational stress among nurses: A cross-sectional study	No nursing shifts reported
2016, Stanojevic C	Health effects of sleep deprivation on nurses working shifts	Study desing: review
2022, Hwang S	Factors Associated with Gastrointestinal Symptoms among Rotating Shift Nurses in South Korea: A Cross-Sectional Study	No nursing shifts reported
2000, Estryn B	Night work in hospital with 8-hour, 10-hour or 12-hour nights	No association between gastrointestinal disorders and sifhts reported

Supplementary Table S6. Funnel of gastrointestinal problems in Daytime vs. Rotating Shifts

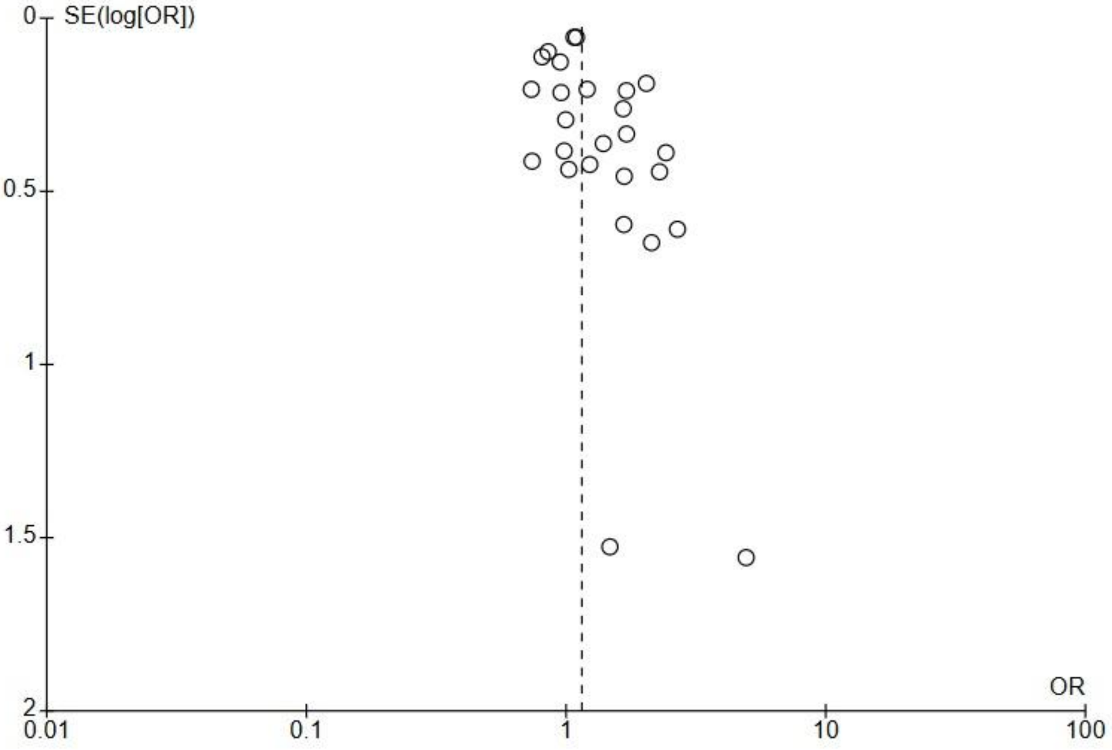

Supplement: Supplementary file 2 [file Table_1.pdf]
